# Prognostic value of cardiopulmonary exercise testing in pulmonary arterial hypertension

**DOI:** 10.1183/13993003.02026-2024

**Published:** 2025-08-21

**Authors:** Andrea Baccelli, Rocco F. Rinaldo, Gulammehdi Haji, Rachel J. Davies, Francesco Lo Giudice, Wendy Gin-Sing, Beatrice Vigo, Stefano Centanni, J. Simon R. Gibbs, Luke S. Howard

**Affiliations:** 1 Department of Respiratory Medicine, Royal Brompton Hospital, Guy's and St Thomas’ NHS Foundation Trust, London, UK; 2 Respiratory Diseases Unit, AOU Città della Salute e della Scienza di Torino, Molinette Hospital, Department of Medical Sciences, University of Turin, Turin, Italy; 3 National Pulmonary Hypertension Service, Hammersmith Hospital, Imperial College Healthcare NHS Trust, London, UK; 4 Respiratory Unit, Azienda Ospedaliera Universitaria San Luigi Gonzaga, Orbassano, Italy; 5 Respiratory Unit, ASST Santi Paolo e Carlo, Department of Health Sciences, Università degli Studi di Milano, Milan, Italy; 6 National Heart and Lung Institute, Imperial College London, London, UK

## Abstract

**Background:**

Current guidelines recommend a four-strata model based on World Health Organization Functional Class (WHO FC), 6-min walk distance (6MWD) and serum levels of brain natriuretic peptide (BNP) or N-terminal pro-BNP (NT-proBNP) for risk stratification in patients with pulmonary arterial hypertension (PAH) during follow-up. We explored the relevance of using cardiopulmonary exercise testing (CPET) as the exercise parameter in place of 6MWD at first reassessment after treatment initiation in PAH.

**Methods:**

Incident treatment-naive patients with idiopathic, heritable, drug/toxin-induced and connective tissue disease-associated PAH between 2010 and 2022 were analysed. Correlations between CPET and haemodynamic and right ventricular function parameters were explored, and those which were significant were carried forward to assess association with survival. Independent predictors were used to derive a four-strata CPET score.

**Results:**

262 patients were included. CPET parameters showed better correlations with haemodynamics and right ventricular function than 6MWD. The CPET score included peak oxygen uptake (peak *V̇*_O_2__), the slope relating minute ventilation to carbon dioxide production (*V̇*_E_/*V̇*_CO_2__ slope) and peak oxygen pulse. The four-strata model based on WHO FC, BNP and CPET score predicted survival at the time of the first re-evaluation, with better accuracy than the model including 6MWD (C-index 0.81 *versus* 0.71). The CPET score on its own also performed well (C-index 0.82) with a greater spread between categories. Treatment-associated changes in peak *V̇*_O_2__ predicted survival, while changes in 6MWD did not.

**Conclusions:**

A simplified four-strata CPET score either alone or included with BNP and WHO FC accurately predicts survival at follow-up in PAH.

## Shareable abstract

**Cardiopulmonary exercise testing reflects underlying changes in haemodynamics and right ventricular function in PAH. A four-strata risk stratification model based on WHO FC, BNP and CPET score accurately predicts survival.** https://bit.ly/4c0yo7F

## Graphical abstract

**Figure:**
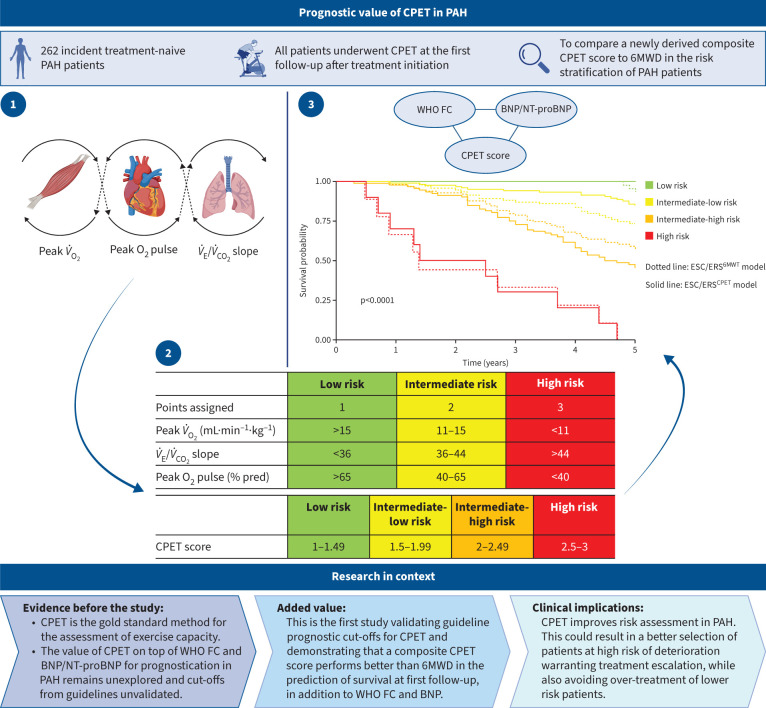
A four-strata risk stratification model that includes World Health Organization Functional Class (WHO FC), brain natriuretic peptide (BNP) or N-terminal pro-BNP (NT-proBNP) and cardiopulmonary exercise testing (CPET) score accurately predicts survival in pulmonary arterial hypertension (PAH). *V̇*_O_2__: oxygen uptake; *V̇*_E_/*V̇*_CO_2__: minute ventilation/carbon dioxide production.

## Introduction

The importance of periodic risk assessment in patients with pulmonary arterial hypertension (PAH) has been highlighted by the latest 2022 European Society of Cardiology/European Respiratory Society (ESC/ERS) guidelines on the diagnosis and management of pulmonary hypertension, with the addition of a four-strata risk score at follow-up to guide treatment decisions on a more granular level [1].

Over time, multiple invasive and non-invasive prognostic parameters have been tested and incorporated into risk assessment tools [2–10]. The simplified four-strata prognostic model (low, intermediate-low, intermediate-high and high risk) based on World Health Organization Functional Class (WHO FC), 6-min walk distance (6MWD) and serum levels of brain natriuretic peptide (BNP) or N-terminal pro-BNP (NT-proBNP) introduced by the guidelines predicts survival in patients with PAH, with observed 1-year mortality rates of 0–3%, 2–7%, 9–19% and >20%, respectively [5].

Cardiopulmonary exercise testing (CPET) represents the gold standard method for the assessment of exercise capacity. It is a non-invasive metabolic test that integrates ventilatory and cardiovascular parameters, providing a comprehensive analysis of exercise limitation. As such, CPET is able to shed light on the pathophysiological mechanisms underlying response to therapy in PAH, unlike the simpler and more widely used 6-min walk test (6MWT) [11–13].

In recent years, a limited, yet growing, body of evidence has highlighted the prognostic relevance of multiple candidate CPET parameters [14–21]. However, the added value of CPET on top of the other commonly used clinical variables used for prognostication in PAH remains unexplored.

The main objective of the present study was to determine the additional prognostic value of CPET variables at first follow-up in PAH and validate the current ESC/ERS cut-offs. Because of the importance of haemodynamics in risk assessment, we sought to: 1) understand the relationships between key CPET variables and prognostic invasive haemodynamic and non-invasive right ventricular function parameters; 2) explore the changes in CPET variables after start of therapy and relationship with mortality; 3) create a CPET score based on independent CPET predictors of mortality which would provide a single number integrating independent CPET variables; and 4) compare a four-strata model incorporating this CPET score as the exercise variable to the currently recommended risk stratification model that includes 6MWD.

## Methods

### Study design

Consecutive incident patients from the National Pulmonary Hypertension Service at Hammersmith Hospital (London, UK) were included prospectively into the TRIPHIC database which was approved under Research Ethics Committee number 17/LO/0563. Demographic, clinical, biochemical, haemodynamic, radiological and functional data were collected and anonymised prior to analysis. The dataset as of 1 January 2023 was analysed.

### Patients

Patients were selected based on the following inclusion criteria: 1) treatment-naive patients aged ≥18 years diagnosed with idiopathic, heritable, drug/toxin-induced, HIV-associated or connective tissue disease-associated PAH between January 2010 and January 2022 based on contemporaneous haemodynamic criteria and the ESC/ERS classification; and 2) first reassessment after treatment initiation within 1 year from diagnosis, with 6MWT and CPET being performed on separate, consecutive days All other forms of pulmonary hypertension were excluded. Further exclusion criteria were death before first reassessment and lack of CPET, BNP or 6MWD at follow-up.

### Right heart catheterisation

All patients underwent a baseline diagnostic right heart catheterisation (RHC). Haemodynamic measurements included right atrial pressure (RAP), systolic, diastolic and mean pulmonary arterial pressure (mPAP), and pulmonary arterial wedge pressure (PAWP). Cardiac output was measured by thermodilution or by the direct Fick method. Cardiac index was calculated as cardiac output/body surface area. Pulmonary vascular resistance (PVR) was calculated as (mPAP−PAWP)/cardiac output. Pulmonary artery blood samples were collected to measure mixed venous oxygen saturation (*S*_vO_2__). Haemodynamic data of the study population are presented in table 1.

**TABLE 1:** Baseline demographic, clinical and functional characteristics of the study group (n=262)

**Age at diagnosis (years)**	54±16
**Female**	162 (62)
**BMI (kg·m^−2^)**	27 (23–31)
**PAH aetiology**	
Idiopathic	167 (64)
CTD-associated	67 (26)
Heritable	13 (5)
Drug/toxin-induced	9 (3)
HIV-associated	6 (2)
**WHO FC**	
I	1 (0.4)
II	25 (10)
III	193 (74)
IV	43 (16)
**BNP (ng·L^−1^)**	234 (73–515)
**6MWD (m)**	269±142
**Acute vasodilator responders**	17 (6)
**Comorbidities**	
Smoking status	
Active smoker	42 (16)
Ex-smoker	81 (31)
Never-smoker	139 (53)
Smoking history (pack-years)	22±18
Coronary artery disease	34 (13)
Systemic hypertension	96 (37)
Diabetes mellitus	50 (19)
Atrial fibrillation	18 (7)
Obesity	81 (31)
Asthma	22 (8)
COPD	26 (10)
Interstitial lung disease	19 (7)
Chronic kidney disease	21 (8)
Thyroid disease	35 (13)
**Haemodynamics**	
Mean systemic BP at RHC (mmHg)	95±16
Hb at RHC (g·dL^−1^)	14.6±2.1
Mean RAP (mmHg)	9±5
Mean PAP (mmHg)	49±13
Mean PAWP (mmHg)	10±4
Cardiac index (L·min^−1^·m^−2^)	2.2±0.7
PVR (WU)	10.2 (6.5–14.7)
*S*_vO_2__ (%)	65±9

Data are presented as mean±sd, n (%) or median (interquartile range). BMI: mass index; PAH: pulmonary arterial hypertension; CTD: connective tissue disease; WHO FC: World Health Organization Functional Class; BNP: brain natriuretic peptide; 6MWD: 6-min walk distance; BP: blood pressure; RHC: right heart catheterisation; Hb: haemoglobin; RAP: right atrial pressure; PAP: pulmonary arterial pressure; PAWP: pulmonary arterial wedge pressure; PVR: pulmonary vascular resistance; *S*_vO_2__: mixed venous oxygen saturation.

### Cardiopulmonary exercise testing

Symptom-limited, incremental maximal CPET using a standard metabolic cart (CPX; Vyaire Medical, Basingstoke, UK) in the upright position was performed on an electromagnetically braked cycle-ergometer (Ergoline, Bitz, Germany) according to American Thoracic Society guidelines and latest recommendations [22, 23]. Oxygen pulse at peak was computed as oxygen uptake (*V̇*_O_2__)/heart rate. Ventilatory efficiency was determined by the minute ventilation/carbon dioxide production (*V̇*_E_/*V̇*_CO_2__) slope excluding resting measures up to the point of the respiratory compensation point.

### Risk stratification

In line with the most recent recommendations, a four-strata approach was adopted. Cut-off levels for WHO FC, BNP and 6MWD were taken from the 2022 ESC/ERS pulmonary hypertension guidelines [1]. A score of 1 was assigned for each parameter in low risk, 2 for intermediate-low risk, 3 for intermediate-high risk and 4 for high risk values, then an average was calculated for each patient, rounded to the nearest integer (supplementary table S1). When deriving a CPET score, a similar approach was taken with cut-offs for already established markers being used (*V̇*_O_2__ and *V̇*_E_/*V̇*_CO_2__ slope) as well as any new independent markers being adopted. To split the intermediate category to develop a four-strata CPET score, we followed the methodology from Kylhammar *et al.* [7] to create an intermediate-low CPET score (1.5–1.99) and an intermediate-high CPET score (2–2.49).

### Statistics

Normality was assessed through the Kolmogorov–Smirnov test. Quantitative data are described as mean and standard deviation or median and interquartile range (IQR) according to their distribution, and qualitative data are described as absolute frequency and percentage. Missing data were not imputed. Patients who underwent lung transplantation were censored on the date of transplantation. Survival time was calculated from the date of diagnostic RHC until death. Transplantation-free survival was analysed with Kaplan–Meier analysis and the log-rank test. Survival time was calculated from the date of diagnostic RHC until death or last recorded clinical contact. Cox proportional hazards regression was used to assess the association between individual exercise parameters/risk category and survival, expressed as hazard ratio and 95% confidence interval. A theory-driven model selection of covariates was adopted, with candidate variables chosen because of their known prognostic relevance and pathophysiological correlations to strong haemodynamic risk factors. The multivariable model was then performed without a stepwise selection of the covariates. For those parameters that have previously been proposed as predictors with published cut-offs, we used these. For new parameters (*e.g.* peak oxygen pulse), time-dependent receiver operating characteristic (ROC) analysis was used to determine the area under the curve (AUC), and optimal thresholds were determined by the value maximising the sum of sensitivity and specificity (Youden index).

Time-dependent ROC analyses of the prognostic models were performed and compared using the DeLong test. Harrell's C-statistic was used to compare accuracy and discrimination of the two risk stratification methods. To further strengthen our findings, the Akaike Information Criterion (AIC) was used to ensure that improved discrimination was not achieved at the cost of excessive model complexity. Lastly, the time-point considered for the AUC analysis was 5 years. Correlation coefficients between exercise parameters and other variables were determined by Spearman's rank correlation analysis. A p-value <0.05 was considered statistically significant. Statistical tests were performed using SPSS Statistics version 28.0 (IBM, Armonk, NY, USA) and Stata version 18 (StataCorp, College Station, TX, USA).

## Results

### Baseline characteristic and survival

**FIGURE 1:**
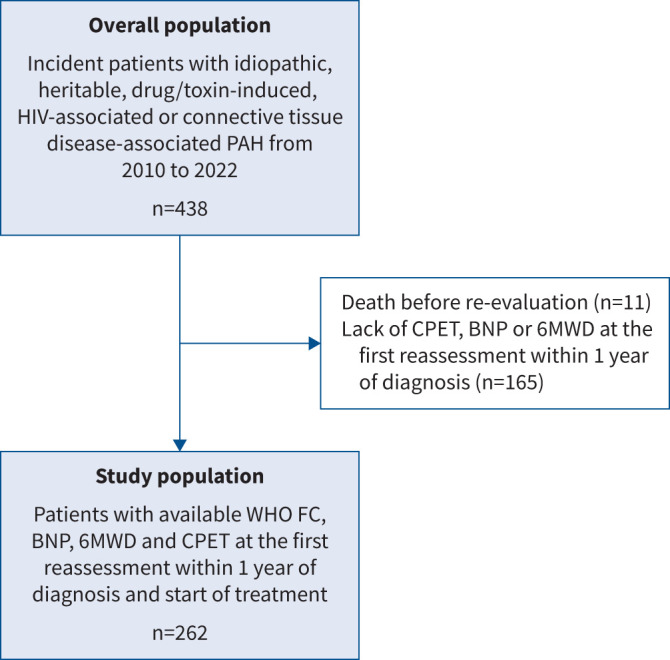
Flow diagram showing the study population and excluded patients. PAH: pulmonary arterial hypertension; CPET: cardiopulmonary exercise testing; BNP: brain natriuretic peptide; 6MWD, 6-min walk distance; WHO FC: World Health Organization Functional Class.

262 patients were included in the final analysis (figure 1). Baseline characteristics are shown in table 1 and supplementary table S2. Patients included in the analysis did not significantly differ from the overall population (n=438), as shown in supplementary table S3. 153 (58%) patients were receiving monotherapy at the time of the first reassessment, while 42% of patients were on combination therapy, including 14 on parenteral prostacyclin analogues (supplementary table S4). The median (IQR) follow-up time was 5.1 (3.6–8.1) years, with 13 years being the longest duration. During follow-up, 120 patients died and two underwent lung transplantation. For the study population, the Kaplan–Meier estimated survival rates 1, 3 and 5 years after diagnosis were 96%, 83% and 65%, respectively. For the overall population, the Kaplan–Meier estimated survival rates 1, 3 and 5 years after diagnosis were 92%, 78% and 61%, respectively.

### CPET variables show stronger correlations with resting haemodynamics and right ventricular function

**FIGURE 2:**
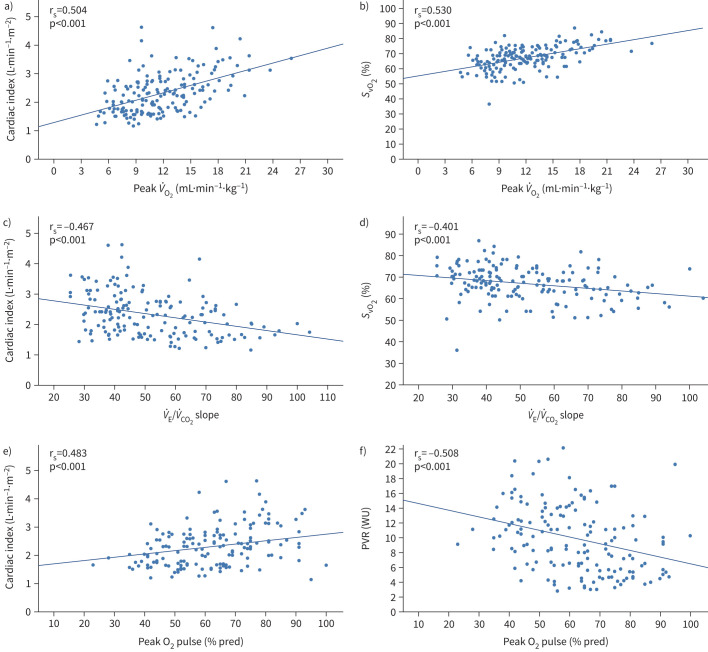
Relations between cardiopulmonary exercise testing parameters and haemodynamic variables: a) correlation between peak oxygen uptake (*V̇*_O_2__) and cardiac index, b) correlation between peak *V̇*_O_2__ and mixed venous oxygen saturation (*S*_vO_2__), c) correlation between minute ventilation/carbon dioxide production (*V̇*_E_/*V̇*_CO_2__) slope and cardiac index, d) correlation between *V̇*_E_/*V̇*_CO_2__ slope and *S*_vO_2__, e) correlation between oxygen pulse and cardiac index, and f) correlation between oxygen pulse and pulmonary vascular resistance (PVR).

Overall, CPET showed more and stronger correlations with invasive haemodynamics and right ventricular function on cardiac magnetic resonance (CMR). Peak *V̇*_O_2__ (mL·min**^−^**^1^·kg^−1^) significantly correlated with prognostically relevant haemodynamic parameters, such as mean RAP (mRAP), cardiac index and *S*_vO_2__, as shown in figure 2 and supplementary table S5. *V̇*_E_/*V̇*_CO_2__ slope displayed significant correlations with cardiac index, *S*_vO_2__ and PVR. Peak oxygen pulse was the CPET variable with the highest number of significant correlations, the strongest ones being with PVR and cardiac index. 6MWD had significant correlations with mRAP, cardiac index, PVR and *S*_vO_2__, the strongest one being with *S*_vO_2__ (r_s_=0.447, p<0.001), as shown in supplementary table S5. All examined CPET variables exhibited a significant correlation with right ventricular ejection fraction (RVEF), as assessed by CMR, with the strongest relation involving peak oxygen pulse (r_s_=0.562, p<0.001), as illustrated in supplementary figure S1 and supplementary table S6. 6MWD did not display a significant correlation with RVEF (p=0.14). Based on these data, we considered absolute *V̇*_O_2__, *V̇*_E_/*V̇*_CO_2__ slope and oxygen pulse % pred to be the strongest correlates from CPET with recognised predictors of survival but continued to present data for *V̇*_O_2__ % pred and absolute oxygen pulse for completeness.

### Survival is associated with changes in CPET after treatment and not 6MWD or BNP

Baseline and follow-up clinical characteristics of patients stratified according to survival status at the end of the observation period are reported in table 2, as well as changes in CPET variables in the subset of patients with available exercise testing at both time-points (n=198). Patients alive at the end of follow-up displayed a significantly higher 6MWD and lower BNP both at baseline and at the first re-evaluation, but with comparable mean changes after start of therapy. Survivors were characterised by a significantly greater improvement in peak *V̇*_O_2__, ventilatory efficiency and peak oxygen pulse % pred than non-survivors.

**TABLE 2:** Baseline and follow-up demographic, clinical and functional characteristics of the study population stratified by survival

	Survivors (n=142)	Non-survivors (n=120)	p-value
**Age at diagnosis (years)**	47±15	62±14	<0.001
**Female**	97 (68)	65 (55)	0.028
**PAH aetiology**			0.002
Idiopathic	98 (59)	69 (41)	
CTD-associated	25 (37)	42 (63)	
Heritable	11 (86)	2 (14)	
Drug/toxin-associated	5 (56)	4 (44)	
HIV-associated	3 (50)	3 (50)	
**Comorbidities**			
Coronary artery disease	11	23	0.005
Systemic hypertension	38	58	<0.001
Diabetes mellitus	18	32	0.003
Atrial fibrillation	8	10	0.359
Obesity	44	37	0.919
Chronic kidney disease	3	18	<0.001
**WHO FC baseline**			0.042
I	1	0	
II	20	5	
III	98	95	
IV	23	20	
**WHO FC reassessment**			<0.001
I	13	1	
II	63	17	
III	65	98	
IV	1	4	
**BNP baseline (ng·L^−1^)**	198 (48–381)	287 (96–742)	<0.001
**BNP reassessment (ng·L^−1^)**	51 (23–90)	156 (71–370)	<0.001
**6MWD baseline (m)**	322±134	206±125	<0.001
**6MWD reassessment (m)**	379±128	263±131	<0.001
**CEPT^#^**			
Peak *V̇*_O_2__ baseline (mL·min**^−^**^1^·kg^−1^)	13.2±4	10.3±3.3	<0.001
Peak *V̇*_O_2__ reassessment (mL·min**^−^**^1^·kg^−1^)	15.5±4.6	11.1±3.8	<0.001
Peak *V̇*_O_2__ baseline (% pred)	52±14	47±13	0.008
Peak *V̇*_O_2__ reassessment (% pred)	62±16	51±15	<0.001
*V̇*_E_/*V̇*_CO_2__ slope baseline	48.9±16.9	57.6±18.5	0.001
*V̇*_E_/*V̇*_CO_2__ slope reassessment	41.2±9.8	53.5±17.2	<0.001
Peak O_2_ pulse baseline (mL·beat**^−^**^1^)	8.7±3.4	7.9±2.8	0.007
Peak O_2_ pulse reassessment (mL·beat**^−^**^1^)	9.9±4.2	9.6±4.3	0.531
Peak O_2_ pulse baseline (% pred)	64±16	63±21	0.852
Peak O_2_ pulse reassessment (% pred)	72±18	63±21	<0.001
**Changes after treatment initiation**			
Δ 6MWD (m)	67±103	53±87	0.322
Δ BNP (ng·L^−1^)	−201±283	−204±506	0.949
Δ Peak *V̇*_O_2__ (mL·min^−1^·kg^−1^)	2.7±3.2 (n=108)	0.6±2.4 (n=90)	<0.001
Δ Peak *V̇*_O_2__ (% pred)	12±12 (n=108)	2±11 (n=90)	<0.001
Δ *V̇*_E_/*V̇*_CO_2__ slope	−8.3±12.8 (n=108)	−2.9±14.2 (n=90)	0.008
Δ Peak O_2_ pulse (mL·beat**^−^**^1^)	2.1±3.2 (n=108)	1.7±3.5 (n=90)	0.062
Δ Peak O_2_ pulse (% pred)	9.2±14.9 (n=108)	−0.4±12.7 (n=90)	<0.001

Data are presented as mean±sd, n (%), n or median (interquartile range). PAH: pulmonary arterial hypertension; CTD: connective tissue disease; WHO FC: World Health Organization Functional Class; BNP: brain natriuretic peptide; 6MWD: 6-min walk distance; CPET: cardiopulmonary exercise testing; *V̇*_O_2__: oxygen uptake; *V̇*_E_/*V̇*_CO_2__: minute ventilation/carbon dioxide production; Δ: change. ^#^: baseline data availability: survivors n=108 and non-survivors n=90.

Exploring the prognostic value of treatment-associated changes in exercise parameters, only changes in peak *V̇*_O_2__ (mL·min**^−^**^1^·kg^−1^) were associated with survival at multivariate Cox regression analysis, as shown in supplementary table S7.

### CPET alone at follow-up predicts survival

Among the CPET variables considered for prognostication in PAH by the guidelines (peak *V̇*_O_2__, both as % pred and weight-adjusted absolute value, and *V̇*_E_/*V̇*_CO_2__ slope) plus oxygen pulse as the additional parameter hereby investigated, only peak *V̇*_O_2__ (mL·min**^−^**^1^·kg^−1^), peak oxygen pulse % pred and *V̇*_E_/*V̇*_CO_2__ slope emerged as independent predictors of survival in multivariate Cox regression analysis (table 3). Based on cut-offs derived from the study population (40% and 65%), peak oxygen pulse % pred was able to significantly stratify survival in this population, as shown in supplementary figure S2. Using values from the ESC/ERS guidelines for peak *V̇*_O_2__ and *V̇*_E_/*V̇*_CO_2__ slope, we included peak oxygen pulse % pred and were thus able to derive a new standalone CPET score (table 4).

**TABLE 3:** Univariate and multivariate Cox regression analysis of cardiopulmonary exercise testing parameters assessed at the first re-evaluation

	Univariate		Multivariate	
	Hazard ratio (95% CI)	p-value	Hazard ratio (95% CI)	p-value
**Peak *V̇*_O_2__ (mL·min^−1^·kg^−1^)**	0.75 (0.70–0.81)	**<0.001**	0.78 (0.75–0.89)	**<0.001**
**Peak *V̇*_O_2__ (% pred)**	0.94 (0.93–0.96)	**<0.001**		
***V̇*_E_/*V̇*_CO_2__ slope**	1.05 (1.04–1.06)	**<0.001**	1.03 (1.02–1.06)	**<0.001**
**Peak O_2_ pulse (mL·beat^−1^)**	0.99 (0.93–1.04)	0.605		
**Peak O_2_ pulse (% pred)**	0.97 (0.96–0.98)	**<0.001**	0.96 (0.94–0.98)	**<0.001**

*V̇*_O_2__: oxygen uptake; *V̇*_E_/*V̇*_CO_2__: minute ventilation/carbon dioxide production. Bold indicates significantly different.

**TABLE 4:** Cardiopulmonary exercise testing variables and cut-off values used for standalone CPET score

	Low risk	Intermediate risk	High risk
**Points assigned**	1	2	3
**Peak *V̇*_O_2__ (mL·min^−1^·kg^−1^)**	>15	11–15	<11
***V̇*_E_/*V̇*_CO_2__ slope**	<36	36–44	>44
**Peak O_2_ pulse (% pred)**	>65	40–65	<40

	Low risk	Intermediate-low risk	Intermediate-high risk	High risk
**CPET score**	1–1.49	1.5–1.99	2–2.49	2.5–3

*V̇*_O_2__: oxygen uptake; *V̇*_E_/*V̇*_CO_2__: minute ventilation/carbon dioxide.

**FIGURE 3:**
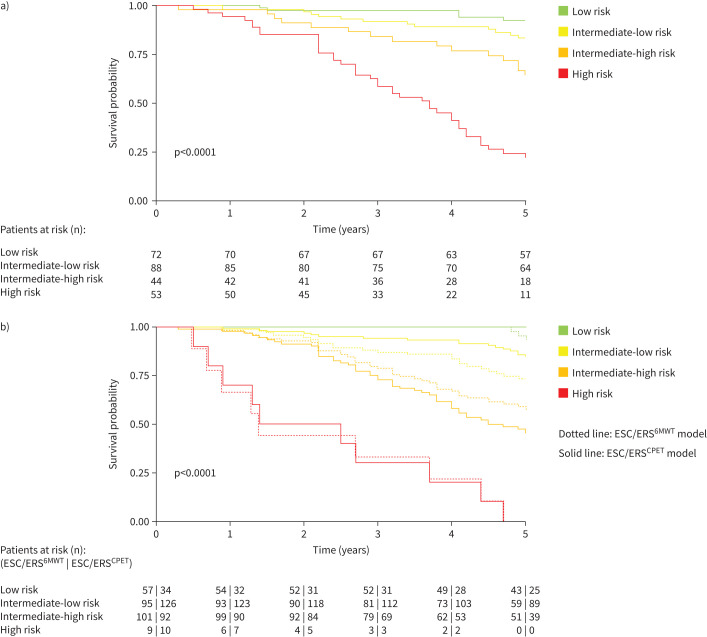
Kaplan–Meier survival curves at first follow-up. a) Transplant-free survival according to the four-strata cardiopulmonary exercise testing (CPET) score risk groups at follow-up. b) Transplant-free survival according to the four-strata ESC/ERS^6MWT^ and ESC/ERS^CPET^ risk categories at follow-up. ESC/ERS: European Society of Cardiology/European Respiratory Society; 6MWT: 6-min walk test.

Using the four-strata CPET score (1–1.49, 1.5–1.99, 2–2.49 and 2.5–3) in isolation, there was an even spread between categories, with 72 patients in the low risk group, 88 in the intermediate-low risk group, 44 in the intermediate-high risk group and 53 in the high risk group. Survival rates are shown in figure 3a (log-rank test p<0.001 for all group comparisons; C-index 0.82).

### CPET score performs better in place of 6MWD in the ESC/ERS four-strata risk model

A four-strata risk stratification model based on WHO FC, BNP and CPET score (ESC/ERS^CPET^) was applied to the study population, based on the cut-offs illustrated in supplementary table S1, obtaining a clear and statistically significant separation of mortality risk between each stratum. The Kaplan–Meier estimated transplant-free survival rates 3 and 5 years after diagnosis for the low risk at first follow-up group were 100% and 100%, respectively; for the intermediate-low risk group 93% and 84%, respectively; for the intermediate-high risk group 62% and 43%, respectively; and for the high risk group 20% and 0%, respectively (log-rank test p<0.0001 for all group comparisons) (figure 3b). Although the patient numbers become smaller, we show excellent discrimination out to 10 years and 100% survival in the ESC/ERS^CPET^ low risk group, representing a truly very low risk group with excellent prognosis, of which 10 (29%) were treated exclusively with calcium channel blockers (supplementary figures S5 and S6).

To show our cohort and dataset align with published literature, we also produced the recommended four-strata model based on WHO FC, BNP and 6MWD (ESC/ERS^6MWT^). The Kaplan–Meier estimated transplant-free survival rates 3 and 5 years after diagnosis for the low risk at first follow-up group were 100% and 91%, respectively; for the intermediate-low risk group 86% and 74%, respectively; for the intermediate-high risk group 68% and 55%, respectively; and for the high risk group 22% and 0%, respectively (log-rank test p<0.0001 for all group comparisons) (figure 3b).

Applying the “French system” of counting the number of low risk criteria, both models including either CPET score or 6MWD were significantly associated with survival at univariate and multivariate Cox regression analysis (supplementary tables S8 and S9), and the Kaplan–Meier transplant-free survival curves according to the number of low risk criteria achieved at first follow-up are shown in supplementary figures S3 and S4 (log-rank test p<0.0001 for all group comparisons).

Discrepancy in the risk category distribution between the ESC/ERS^6MWT^ and ESC/ERS^CPET^ strata risk models was observed in 68 patients (26%; κ=0.61, 95% CI 0.58–0.65), as shown in supplementary table S10. In discordant cases, a significantly lower survival probability was found when the CPET score grade was higher than the 6MWD score (p<0.001).

**FIGURE 4:**
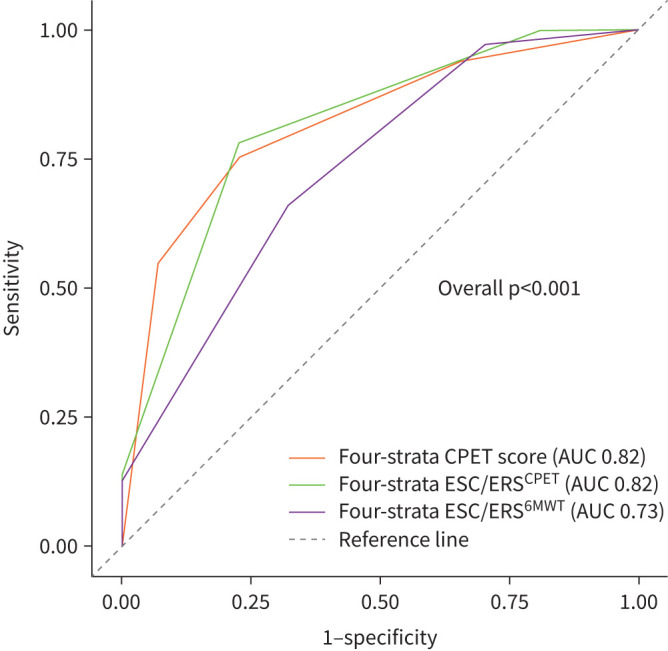
Receiver operating characteristic curves and areas under the curve (AUC) of the cardiopulmonary exercise testing (CPET) score, ESC/ERS^6MWT^ and ESC/ERS^CPET^ risk stratification models. The AUC for the CPET score was 0.82 (95% CI 0.77–0.86), for the ESC/ERS^CPET^ was 0.82 (95% CI 0.78–0.86) and for the ESC/ERS^6MWT^ was 0.73 (95% CI 0.69–0.77). ESC/ERS: European Society of Cardiology/European Respiratory Society; 6MWT: 6-min walk test.

Time-dependent ROC analysis and differences in concordance statistics were used to compare stratification strategies at 5 years. The ESC/ERS^CPET^ Cox model had a significantly higher C-index than the ESC/ERS^6MWT^ (0.81 (95% CI 0.75–0.87) *versus* 0.71 (95% CI 0.64–0.78); p<0.001). The ESC/ERS^CPET^ Cox model had a higher AUC than the ESC/ERS^6MWT^ (0.82 (95% CI 0.78–0.86) *versus* 0.73 (95% CI 0.69–0.77); p<0.001). ROC curves are shown in figure 4. The AIC values were 712 for ESC/ERS^CPET^ and 698 for ESC/ERS^6MWT^.

### Comparison of both scores in patients with and without cardiopulmonary comorbidities

The predictive performance of ESC/ERS^CPET^ and ESC/ERS^6MWT^ was also tested in the subgroups of patients with and without cardiopulmonary comorbidities. These were defined by the presence of at least three risk factors among BMI ≥30 kg·m^−2^, systemic hypertension, diabetes mellitus, coronary artery disease, smoking history and diffusing capacity of the lung for carbon monoxide <45% predicted [1]. In patients without cardiopulmonary comorbidities, the AUC and C-index for ESC/ERS^CPET^ were 0.79 (95% CI 0.7–0.85) and 0.81 (95% CI 0.74–0.83), respectively, and for ESC/ERS^6MWT^ were 0.73 (95% CI 0.69–0.8) and 0.72 (95% CI 0.65–0.84), respectively. In patients with cardiopulmonary comorbidities, the AUC and C-index for ESC/ERS^CPET^ were 0.74 (95% CI 0.67–0.81) and 0.72 (95% CI 0.63–0.83), respectively, and for ESC/ERS^6MWT^ were 0.70 (95% CI 0.69–0.84) and 0.70 (95% CI 0.61–0.79), respectively.

## Discussion

Risk stratification at baseline uses multiple parameters, including haemodynamics and imaging, as well as exercise capacity to guide a relatively straightforward binary decision between dual oral or triple therapy with intravenous prostacyclin [1, 24]. There is, however, inhomogeneity in response to treatment and thus risk stratification at first follow-up is critical for determining the best long-term treatment strategy [3, 5–7, 25–28]. Using the largest cohort to date of patients undergoing CPET following treatment, we tested and verified the hypothesis that a composite CPET score, on its own as well as in addition to WHO FC and BNP, predicts survival in incident PAH patients evaluated within 12 months from treatment initiation.

Rooting our approach in the pathophysiology of the disease, we first demonstrated that absolute *V̇*_O_2__ per kg and *V̇*_E_/*V̇*_CO_2__ slope correlate with haemodynamic and magnetic resonance measures of pulmonary hypertension severity, thus confirming the utility of the 2015/2022 ESC/ERS guideline recommendations for the use of CPET in risk stratification [1, 29]. This also supports the proposed removal of peak *V̇*_O_2__ % pred in the 7th World Symposium of Pulmonary Hypertension (WSPH) expert consensus [28]. Second, we showed that oxygen pulse (a CPET surrogate of stroke volume) had the most correlations with other known predictors of survival.

Next, we showed that changes in these three CPET variables from baseline to first follow-up predict survival in a univariate model, unlike changes in 6MWD and BNP, and then that changes in peak *V̇*_O_2__ and oxygen pulse % pred are independent predictors at multivariate analysis.

Following the 2015 ESC/ERS guidelines, it was recognised that many patients fell into a large intermediate risk group, and further refinement was proposed in the 2022 ESC/ERS guidelines, by dividing the intermediate group in two [1, 29]. Based on our findings, we developed a CPET score and have shown that using this instead of 6MWD provides better discrimination when used in the ESC/ERS four-strata score. Remarkably, we show that using CPET on its own without WHO FC and BNP provides even better discrimination.

The recent 7th WSPH highlighted the benefit of using haemodynamics to discriminate intermediate-low and intermediate-high further into four risk categories [28, 30]. CPET is likely to reflect pulmonary hypertension severity more than 6MWD given its closer relationship with peak cardiac output [11, 31–33]. Cardiac output is the major determinant of peak exercise capacity, assessed by absolute *V̇*_O_2__, whereas maximum cardiac output may not be achieved in many patients undertaking 6MWT, in particular younger patients [12, 22, 31]. In pulmonary hypertension, the major determinant of cardiac output is stroke volume, which is known to be an independent predictor of survival [11, 25, 26, 31, 34]. Oxygen pulse is a surrogate of stroke volume as it relates to the amount of oxygen consumed with each heartbeat [22]. In pulmonary hypertension, oxygen pulse is typically at a plateau in the last few minutes of an incremental exercise test, thus reducing error in measurement and taking out any effort-related component [35]. Relatedly, Badagliacca *et al.* [20] demonstrated that right ventricular fractional area change and oxygen pulse predicted clinical worsening at the time of diagnosis in a group of 130 idiopathic PAH patients. Here we show that oxygen pulse at follow-up as well as its change over time represent strong independent predictors of survival. Importantly, however, oxygen pulse does not simply reflect stroke volume, as it represents the product of stroke volume and arterial–venous difference in the content of oxygen at any given point. In the context of new drugs that potentially increase oxygen content and extraction, without significant increase in resting cardiac output (*e.g.* sotatercept) [36], the assessment of oxygen pulse in conjunction with haemoglobin may provide additional insights into treatment responses.

Similarly, *V̇*_E_/*V̇*_CO_2__ slope is effort independent, since it excludes resting and peak data points [22]. Physiologically and mathematically, it relates to the arterial carbon dioxide set point which reduces in accordance with heart failure severity and the physiological dead-space ventilation which reflects pulmonary vascular disease [13, 37–39]. It is thus a reliably observable composite measure of the severity of cardiopulmonary impairment. Lastly, CPET variables correlated well with measures of right ventricular function on magnetic resonance imaging, unlike 6MWD, so it is not surprising that it performs well in predicting outcomes.

What is particularly thought provoking and impressive is that the standalone CPET score performs better on its own than with the inclusion of WHO FC and BNP. It results in much larger numbers of patients falling into the low and high risk groups, giving clearer guidance on when to stick with current therapy or escalate to more aggressive intravenous therapy. It may be that WHO FC and BNP, particularly the former, dilutes the predictive capacity of CPET.

The main rationale for the four-strata risk score in the ESC/ERS guidelines was to divide up the large amorphous group of intermediate risk patients, by subdividing the cut-offs of the previously used parameters, and this has certainly improved risk stratification. Nonetheless, using this approach, we observe that, as well as still having only a small number of low and high risk patients, CPET in place of 6MWD provides better separation between the intermediate-low and intermediate-high groups (figure 3b). The value of CPET has been confirmed also by Badagliacca *et al*. [19], who showed that stroke volume index (SVI) and peak *V̇*_O_2__ can provide important information to further stratify idiopathic PAH patients who are at intermediate risk after institution of targeted therapies. More recently, peak *V̇*_O_2__ in place of 6MWD resulted in a better discrimination of intermediate-high risk patients in a prevalent PAH cohort from the Spanish registry [40]. We therefore speculate, based on our observations in consecutive patients at first follow-up, that the closer correlation of CPET than 6MWD with haemodynamics and right ventricular function may account for the greater separation of the two categories. This is supported by the recent observation of the added benefit of including SVI and *S*_vO_2__ over the standard ESC/ERS^6MWT^ [30]. This requires invasive testing. Gaining good separation of intermediate-low and intermediate-high is clearly important as the therapeutic decisions are radically different in terms of complexity and it may help guide treatment decisions, which now include sotatercept [24].

There are of course potential limitations in this study which open up the opportunity for further work. This is a single-centre database study, based on consecutively recruited patients. Two of the three measures in the CPET score and their cut-offs were predefined and taken from existing guidelines, then validated in our cohort, but the oxygen pulse cut-offs were derived from our cohort and this requires further external validation. There were also some missing data, but we showed how the overall and study population did not significantly differ, thus reducing the risk of selection bias in this study.

### Conclusion

We have demonstrated the utility of CPET by being the first study to validate the 2015/2022 ESC/ERS guideline prognostic cut-offs and its benefit over 6MWD in terms of physiological correlation with haemodynamics and right ventricular function. It performs better in predicting long-term survival when measured at first follow-up, both in absolute terms and change from baseline, and, in addition to WHO FC and BNP, provides value in separating intermediate-low from intermediate-high groups, which is a major treatment decision point with significant consequences for patients in terms of burden of therapy.

## Shareable PDF

10.1183/13993003.02026-2024.Shareable1 This PDF extract can be shared freely online.
Shareable PDF

ERJ-02026-2024.Shareable

## Supplementary material

**Please note:** supplementary material is not edited by the Editorial Office, and is uploaded as it has been supplied by the author. Supplementary material ERJ-02026-2024.Supplement
