# Supplementary material for: Prognostic value of cardiopulmonary exercise testing in pulmonary arterial hypertension
Source: Eur Respir J. 2025 Aug 21;66(2):2402026. doi: 10.1183/13993003.02026-2024 (PMC12371317; doi:10.1183/13993003.02026-2024)
Supplement: Supplementary file 1 [file ERJ-02026-2024.Shareable.pdf]

SHAREABLE PDF

EUROPEAN RESPIRATORY JOURNAL
ORIGINAL RESEARCH ARTICLE
A. BACCELLI ET AL.

# Prognostic value of cardiopulmonary exercise testing in pulmonary arterial hypertension

Andrea Baccelli, Rocco F. Rinaldo, Gulammehdi Haji, Rachel J. Davies, Francesco Lo Giudice, Wendy Gin-Sing, Beatrice Vigo, Stefano Centanni, J. Simon R. Gibbs and Luke S. Howard

**Prognostic value of CPET in PAH**

262 incident treatment-naive PAH patients

All patients underwent CPET at the first follow-up after treatment initiation

To compare a newly derived composite CPET score to 6MWD in the risk stratification of PAH patients

1

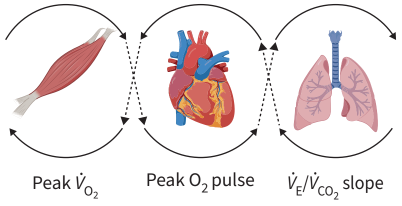


3

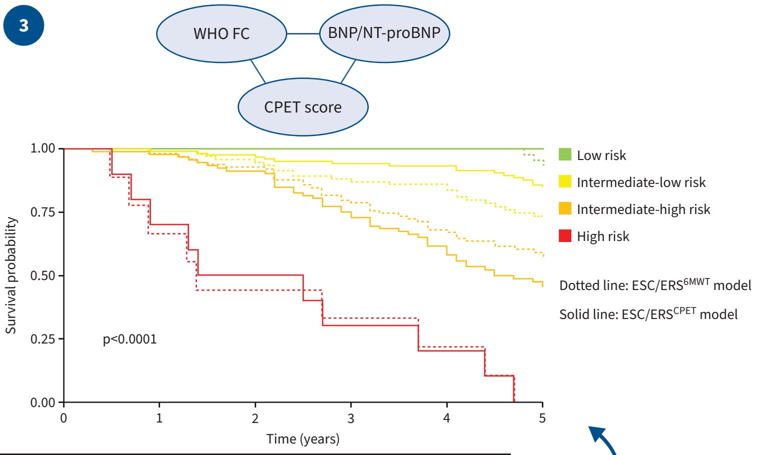


2

| | Low risk | Intermediate risk | High risk |
|---|---|---|---|
| Points assigned | 1 | 2 | 3 |
| Peak $\dot{V}_{O_2}$ (mL·min$^{-1}$·kg$^{-1}$) | >15 | 11–15 | <11 |
| $\dot{V}_E/\dot{V}_{CO_2}$ slope | <36 | 36–44 | >44 |
| Peak $O_2$ pulse (% pred) | >65 | 40–65 | <40 |

| | Low risk | Intermediate-low risk | Intermediate-high risk | High risk |
|---|---|---|---|---|
| CPET score | 1–1.49 | 1.5–1.99 | 2–2.49 | 2.5–3 |

**Research in context**

**Evidence before the study:**
- CPET is the gold standard method for the assessment of exercise capacity.
- The value of CPET on top of WHO FC and BNP/NT-proBNP for prognostication in PAH remains unexplored and cut-offs from guidelines unvalidated.

**Added value:**
This is the first study validating guideline prognostic cut-offs for CPET and demonstrating that a composite CPET score performs better than 6MWD in the prediction of survival at first follow-up, in addition to WHO FC and BNP.

**Clinical implications:**
CPET improves risk assessment in PAH. This could result in a better selection of patients at high risk of deterioration warranting treatment escalation, while also avoiding over-treatment of lower risk patients.

**GRAPHICAL ABSTRACT** A four-strata risk stratification model that includes World Health Organization Functional Class (WHO FC), brain natriuretic peptide (BNP) or N-terminal pro-BNP (NT-proBNP) and cardiopulmonary exercise testing (CPET) score accurately predicts survival in pulmonary arterial hypertension (PAH). $\dot{V}_{O_2}$: oxygen uptake; $\dot{V}_E/\dot{V}_{CO_2}$: minute ventilation/carbon dioxide production.

EUROPEAN RESPIRATORY JOURNAL
ORIGINAL RESEARCH ARTICLE
A. BACCELLI ET AL.

SHAREABLE PDF

# Prognostic value of cardiopulmonary exercise testing in pulmonary arterial hypertension

Andrea Baccelli [1], Rocco F. Rinaldo [2], Gulammehdi Haji[3], Rachel J. Davies[3], Francesco Lo Giudice[3], Wendy Gin-Sing[3], Beatrice Vigo[4], Stefano Centanni[5], J. Simon R. Gibbs[6] and Luke S. Howard [3,6]

[1]Department of Respiratory Medicine, Royal Brompton Hospital, Guy's and St Thomas' NHS Foundation Trust, London, UK. [2]Respiratory Diseases Unit, AOU Città della Salute e della Scienza di Torino, Molinette Hospital, Department of Medical Sciences, University of Turin, Turin, Italy. [3]National Pulmonary Hypertension Service, Hammersmith Hospital, Imperial College Healthcare NHS Trust, London, UK. [4]Respiratory Unit, Azienda Ospedaliera Universitaria San Luigi Gonzaga, Orbassano, Italy. [5]Respiratory Unit, ASST Santi Paolo e Carlo, Department of Health Sciences, Università degli Studi di Milano, Milan, Italy. [6]National Heart and Lung Institute, Imperial College London, London, UK.

Corresponding author: Luke S. Howard (l.howard@imperial.ac.uk)

Check for updates

Shareable abstract (@ERSpublications)
**Cardiopulmonary exercise testing reflects underlying changes in haemodynamics and right ventricular function in PAH. A four-strata risk stratification model based on WHO FC, BNP and CPET score accurately predicts survival.** https://bit.ly/4c0yo7F

**Cite this article as:** Baccelli A, Rinaldo RF, Haji G, *et al*. Prognostic value of cardiopulmonary exercise testing in pulmonary arterial hypertension. *Eur Respir J* 2025; 66: 2402026 [DOI: 10.1183/13993003.02026-2024].

This PDF extract can be shared freely online.

Copyright ©The authors 2025.

This version is distributed under the terms of the Creative Commons Attribution Licence 4.0.

This article has an editorial commentary: https://doi.org/10.1183/13993003.00849-2025

Received: 11 Oct 2024
Accepted: 20 March 2025

## Abstract

***Background*** Current guidelines recommend a four-strata model based on World Health Organization Functional Class (WHO FC), 6-min walk distance (6MWD) and serum levels of brain natriuretic peptide (BNP) or N-terminal pro-BNP (NT-proBNP) for risk stratification in patients with pulmonary arterial hypertension (PAH) during follow-up. We explored the relevance of using cardiopulmonary exercise testing (CPET) as the exercise parameter in place of 6MWD at first reassessment after treatment initiation in PAH.

***Methods*** Incident treatment-naive patients with idiopathic, heritable, drug/toxin-induced and connective tissue disease-associated PAH between 2010 and 2022 were analysed. Correlations between CPET and haemodynamic and right ventricular function parameters were explored, and those which were significant were carried forward to assess association with survival. Independent predictors were used to derive a four-strata CPET score.

***Results*** 262 patients were included. CPET parameters showed better correlations with haemodynamics and right ventricular function than 6MWD. The CPET score included peak oxygen uptake (peak $\dot{V}_{O_2}$), the slope relating minute ventilation to carbon dioxide production ($\dot{V}_E/\dot{V}_{CO_2}$ slope) and peak oxygen pulse. The four-strata model based on WHO FC, BNP and CPET score predicted survival at the time of the first re-evaluation, with better accuracy than the model including 6MWD (C-index 0.81 *versus* 0.71). The CPET score on its own also performed well (C-index 0.82) with a greater spread between categories. Treatment-associated changes in peak $\dot{V}_{O_2}$ predicted survival, while changes in 6MWD did not.

***Conclusions*** A simplified four-strata CPET score either alone or included with BNP and WHO FC accurately predicts survival at follow-up in PAH.
