# Supplementary material for: Prognostic value of cardiopulmonary exercise testing in pulmonary arterial hypertension
Source: Eur Respir J. 2025 Aug 21;66(2):2402026. doi: 10.1183/13993003.02026-2024 (PMC12371317; doi:10.1183/13993003.02026-2024)

Prognostic value of follow-up cardiopulmonary exercise testing in pulmonary arterial hypertension

SUPPLEMENTARY MATERIAL

TABLES

Table S1. Variables and cut-off values used for risk stratification.

Simplified ESC/ERS four-strata risk assessment tool				
	**Low risk**	**Intermediate-low risk**	**Intermediate-high risk**	**High risk**
**Points assigned**	1	2	3	4
**WHO-FC**	I or II	-	III	IV
**BNP (ng/L)**	<50	50-199	200-800	>800
**6MWD (m)**	>440	320-440	165-319	<165
Proposed four-strata risk assessment tool incorporating the CPET score				
	**Low risk**	**Intermediate-low risk**	**Intermediate-high risk**	**High risk**
**Points assigned**	1	2	3	4
**WHO-FC**	I or II	-	III	IV
**BNP (ng/L)**	<50	50-199	200-800	>800
**CPET score**	1-1.49	1.5-1.99	2-2.49	2.5-3

WHO-FC indicates World Health Organization functional class; BNP, Brain natriuretic peptide; 6MWD, 6-minute walking distance; CPET, Cardiopulmonary exercise testing.

Table S2. Baseline echocardiographic, cardiopulmonary exercise testing and pulmonary function test characteristics of the study group.

	**N=262**
Echocardiography (N=262)	
**TAPSE/SPAP, mm/mmHg**	0.225 (0.120)
**RA area, cm^2**	24.1 (7.3)
**Pericardial effusion, n (%)**	35 (14%)
Cardiopulmonary exercise testing (N= 198)	
**Peak VO_2, mL/min/Kg**	11.8 (3.9)
**Peak VO_2, %pred**	50 (14)

**AT, %VO_2max (n=112)**	38 (10)
**Peak work, Watts**	61 (32)
**Peak Work, %predicted**	52 (24)
**VE/VCO_2 slope**	52.9 (18.2)
**VO_2/W slope**	7 (3)
**Peak O_2 pulse, mL/beat**	7.8 (3.2)
**Peak O_2 pulse, %predicted**	64 (19)
**Breathing reserve, %**	34 (18)
**Heart rate reserve, %**	20 (16)
**RER**	1.07 (0.11)
**PetCO_2, KPa**	2.9 (0.8)
**Peak PaCO_2, kPa (n=128)**	4.03 (0.87)
**Dead Space, % (n=125)**	41 (11)
**A-a gradient, kPa (n=122)**	7.46 (2.66)
**Peak Lactate, mmol/L (n=128)**	4.8 (2.1)
**Peak systolic BP, mmHg**	117 (23)
**Peak, diastolic BP, mmHg**	78 (14)
<u>Pulmonary Function Tests (N=262)</u>	
**FEV_1, %predicted**	84 (18)
**FVC, %predicted**	91 (20)
**TLCO, %predicted**	53 (20)
**KCO, %predicted**	65 (22)

All quantitative data: mean (standard deviation), unless otherwise specified. TAPSE indicates tricuspid annular plane systolic excursion; SPAP, systolic pulmonary artery pressure; RA right atrial; VO_2, oxygen consumption; VE/VCO_2 slope, slope relating minute ventilation to carbon dioxide production ratio; VO_2/W slope, slope relating oxygen consumption to workload ratio; RER, respiratory exchange ratio; PetCO_2, end-tidal partial pressure of carbon dioxide; PaCO_2, arterial partial pressure of carbon dioxide; A-a gradient, alveolar to arterial gradient for oxygen; FEV_1, forced expiratory volume in the first second; FVC, forced vital capacity; TLCO, transfer factor of the lung for carbon monoxide; KCO, transfer coefficient of the lung for carbon monoxide.

Table S3. Demographics and baseline characteristics of overall and study populations.

	Overall population (N=438)	Study population (N=262)
**Age, years**	56 (17)	54 (16)
**Female, n (%)**	267 (61%)	162 (62%)
**BMI, kg/m^2 #**	28 (24-31)	27 (23-31)
**PAH aetiology, n (%)**		
**Idiopathic**	276 (63%)	167 (64%)
**CTD-associated**	118 (27%)	67 (26%)
**Heritable**	18 (4%)	13 (5%)
**Drug/Toxins-associated**	13 (3%)	9 (3%)
**HIV-associated**	13 (3%)	6 (2%)
**WHO-FC, I/II/III/IV, n (%)**	2/ 44 (10%)/ 310 (71%)/ 82 (19%)	1/ 25 (10%)/ 193 (74%)/ 43 (16%)
**BNP, ng/L #**	269 (75-619)	234 (73-515)
**6MWD, m**	252 (144)	269 (142)
**Mean RAP, mmHg**	9 (5)	9 (5)
**Mean PAP, mmHg**	50 (13)	49 (13)
**Mean PAWP, mmHg**	10 (4)	10 (4)
**Cardiac index, L/min/m^2**	2.3 (0.7)	2.2 (0.7)
**PVR, WU#**	10.4 (6.5-15.1)	10.2 (6.5-14.7)

All quantitative data: mean (standard deviation), unless otherwise specified. #median (interquartile range). BMI indicates body mass index; CTD, connective tissue disease; WHO-FC, World Health Organization functional class; BNP, Brain natriuretic peptide; 6MWD, 6-minute walking distance; RAP, right atrial pressure; PA, pulmonary artery pressure; PAWP, pulmonary artery wedge pressure; PVR, pulmonary vascular resistance.

Table S4. PAH medications used at the time of the first follow-up visit.

	Study population (N=262)
**Monotherapy**	153
**Combination therapy**	109
**Combination therapy inc. IV/SC PCA**	14

**CCB**	17
**PDE5i**	211
**ERA**	129
**PCA**	14

CCB indicates calcium channel blocker; ERA, endothelin receptor antagonists; PDE5i, phosphodiesterase-5 inhibitors; sGCs, stimulator of soluble guanylate cyclase; IV, intravenous; SC, subcutaneous; PCA, prostacyclin analogues.

Table S5. Relations between exercise variables and hemodynamic variables.

		Peak VO_2 ml/min/Kg	Peak VO_2 %predicted	VE/VCO_2 slope	Peak O_2 pulse %predicted	6MWD
**Mean RAP**	rs	**-.254****	**-.174***	0.106	**-.170***	**-.310****
	p-value	0.001	0.022	0.171	0.026	<0.001
**Mean PAP**	rs	-0.078	**-.256****	0.091	**-.317****	0.043
	p-value	0.302	0.001	0.233	<0.001	0.537
**Cardiac Index**	rs	**.504****	**.380****	**-.467****	**.483****	**.369****
	p-value	<0.001	<0.001	<0.001	<0.001	<0.001
**PVR**	rs	**-.340****	**-.398****	**.326****	**-.508****	**-.171***
	p-value	<0.001	<0.001	<0.001	<0.001	0.015
**SvO_2**	rs	**.530****	**.398****	**-.401****	**.261****	**.447****
	p-value	<0.001	<0.001	<0.001	0.001	<0.001

RAP indicates right atrial pressure; PAP, pulmonary artery pressure; PAWP, pulmonary artery wedge pressure; PVR, pulmonary vascular resistance; SvO_2, mixed venous oxygen saturation; VO_2, oxygen consumption; VE/VCO_2 slope, slope relating minute ventilation to carbon dioxide production ratio; 6MWD, 6-minute walk distance; rs, r squared. ** Correlation is significant at the 0.01 level (2-tailed). * Correlation is significant at the 0.05 level (2-tailed).

Table S6. Relation between exercise variables and RVEF as assessed by cardiac magnetic resonance.

		**Peak VO_2 ml/min/Kg**	**Peak VO_2 %predicted**	**VE/VCO_2 slope**	**Peak O_2 pulse %predicted**	**6MWD**
**RVEF (CMR)**	rs	**.453****	**.348****	**-.320****	**.562****	0.242
	p-value	<0.001	0.001	0.003	<0.001	0.14

RVEF indicates right ventricular ejection fraction; VO_2, oxygen consumption; VE/VCO_2 slope, slope relating minute ventilation to carbon dioxide production ratio; 6MWD, 6-minute walk distance; rs, r squared. ** Correlation is significant at the 0.01 level (2-tailed). * Correlation is significant at the 0.05 level (2-tailed).

Table S7. Univariate and multivariate Cox regression analysis of treatment-associated changes in exercise variables at first re-evaluation.

	Univariate		Multivariate	
	**Hazard ratio (95% CI)**	**p-value**	**Hazard ratio (95% CI)**	**p-value**
**Δ Peak VO_2, ml/min/Kg**	0.83 (0.75-0.92)	**<0.001**	0.98 (0.96-0.99)	**0.024**
**Δ Peak VO_2, %predicted**	0.96 (0.94-0.98)	**<0.001**		
**Δ VE/VCO_2 slope**	1.03 (1.01-1.05)	**0.043**		
**Δ Peak O_2 pulse %predicted**	0.97 (0.96-0.98)	**<0.001**		
**Δ 6MWD, m**	1 (1-1.01)	0.787		

VO_2, oxygen consumption; VE/VCO_2 slope, slope relating minute ventilation to carbon dioxide production ratio; 6MWD, 6-minute walk distance.

Table S8. Univariate and multivariate Cox regression analysis of low-risk criteria assessed at first re-evaluation.

	Univariate		Multivariate	
	**Hazard ratio (95% CI)**	**p-value**	**Hazard ratio (95% CI)**	**p-value**
**WHO-FC I or II**	0.30 (0.16-0.56)	**<0.001**	0.38 (0.22-0.65)	**<0.001**
**BNP <50 ng/L**	0.26 (0.14-0.48)	**<0.001**	0.40 (0.23-0.70)	**0.001**
**CPET score <1.5**	0.16 (0.06-0.38)	**<0.001**	0.39 (0.21-0.73)	**0.003**

WHO-FC indicates World Health Organization functional class; BNP, Brain natriuretic peptide; CPET, Cardiopulmonary exercise testing.

Table S9. Univariate and multivariate Cox regression analysis of low-risk criteria assessed at first re-evaluation.

	Univariate		Multivariate	
	**Hazard ratio (95% CI)**	**p-value**	**Hazard ratio (95% CI)**	**p-value**
**WHO-FC I or II**	0.30 (0.16-0.56)	**<0.001**	0.47 (0.27-0.81)	**0.006**
**BNP <50 ng/L**	0.26 (0.14-0.48)	**<0.001**	0.34 (0.20-0.60)	**<0.001**
**6MWD >440 m**	0.23 (0.09-0.57)	**<0.001**	0.29 (0.14-0.60)	**<0.001**

WHO-FC indicates World Health Organization functional class; BNP, Brain natriuretic peptide; 6MWD, 6-minute walk distance.

Table S10. Risk category distribution between the ESC/ERS four-strata risk stratification model and the newly proposed prognostic tool with the CPET score.

		ESC/ERS^{6MWT}			
		Low	Intermediate-low	Intermediate-high	High
ESC/ERS^{CPET}	Low	**32**	2	-	-
	Intermediate-low	25	**77**	24	-
	Intermediate-high	-	16	**76**	-
	High	-	-	1	**9**

Darker shading indicates CPET score classifies as higher risk than standard model.
Medium shading indicates no change in risk category.
Lighter shading indicates CPET score classifies as lower risk than standard model.

FIGURES

Figure S1. Relation between exercise parameters and right ventricular ejection fraction (RVEF), assessed by cardiac magnetic resonance.

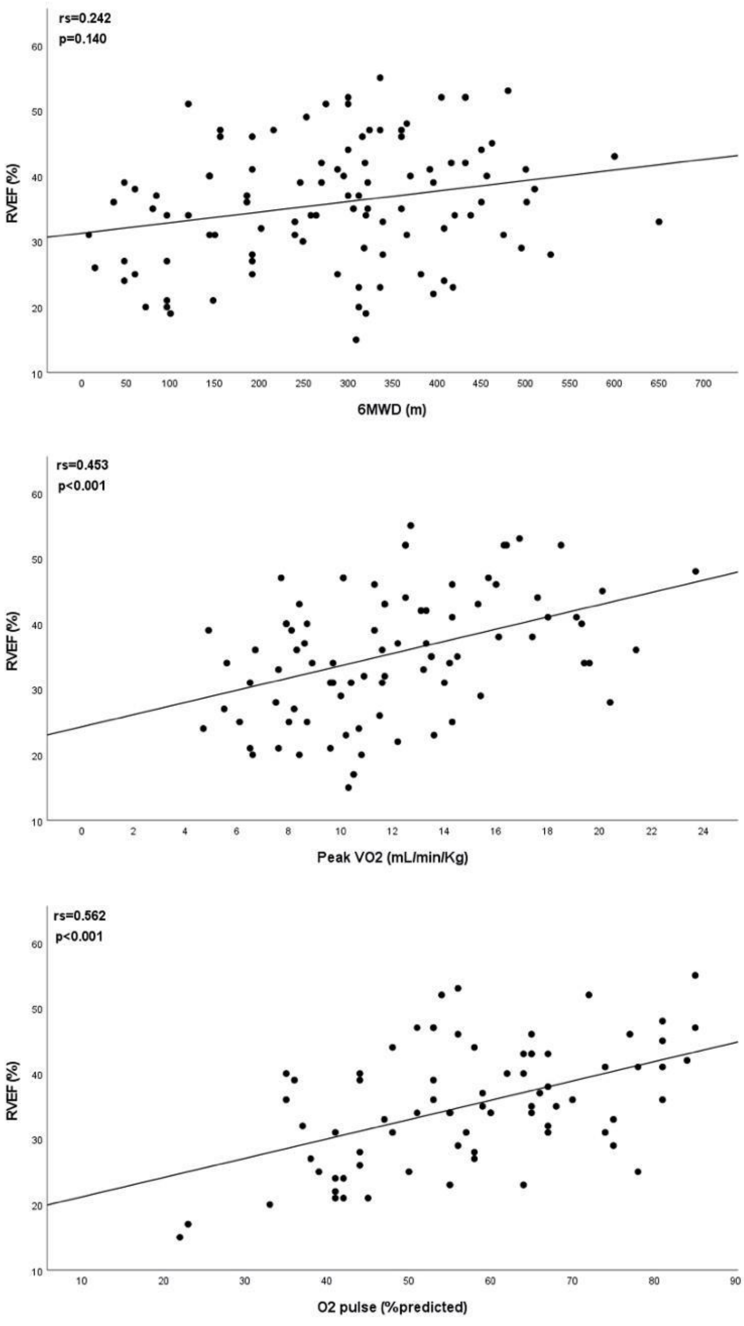

RVEF indicates right ventricular ejection fraction; 6MWD, 6-minute walk distance; VO_2, oxygen consumption.

Figure S2. Transplant-free survival according to the three-strata peak O_2 pulse (%predicted) scores assessed at the first re-evaluation.

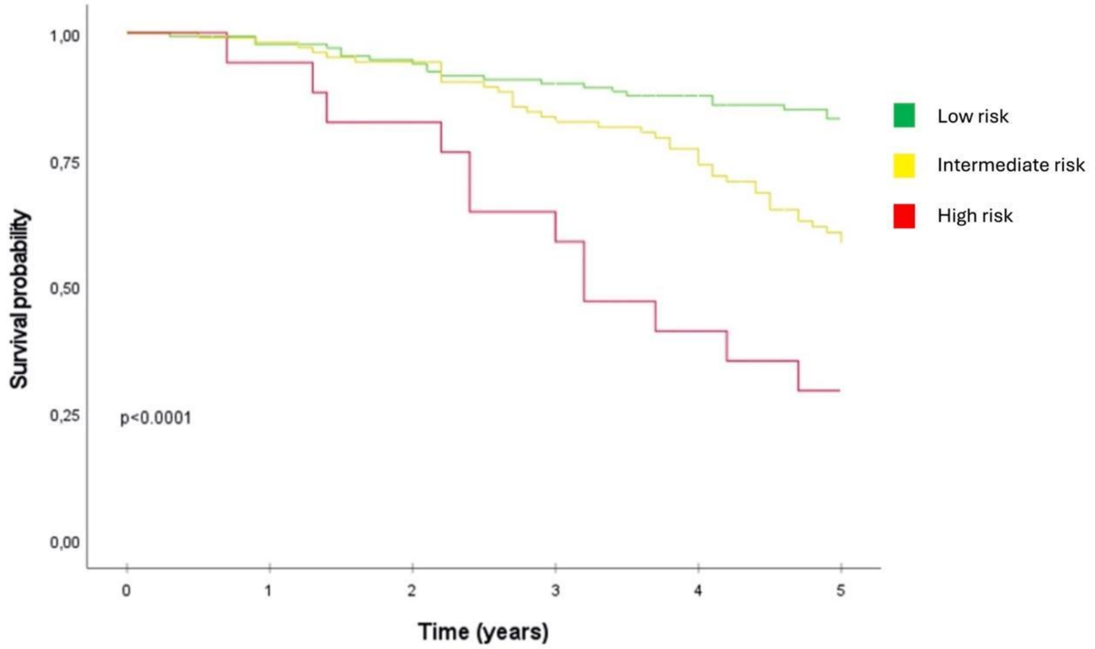

Patients at risk

Low risk	136	129	122	115	104	92
Intermediate risk	104	102	97	85	72	53
High risk	17	16	14	11	7	5

Figure S3. Transplant-free survival according to the number of low-risk criteria (BNP <50 ng/L, WHO FC I-II and a CPET score <1.5).

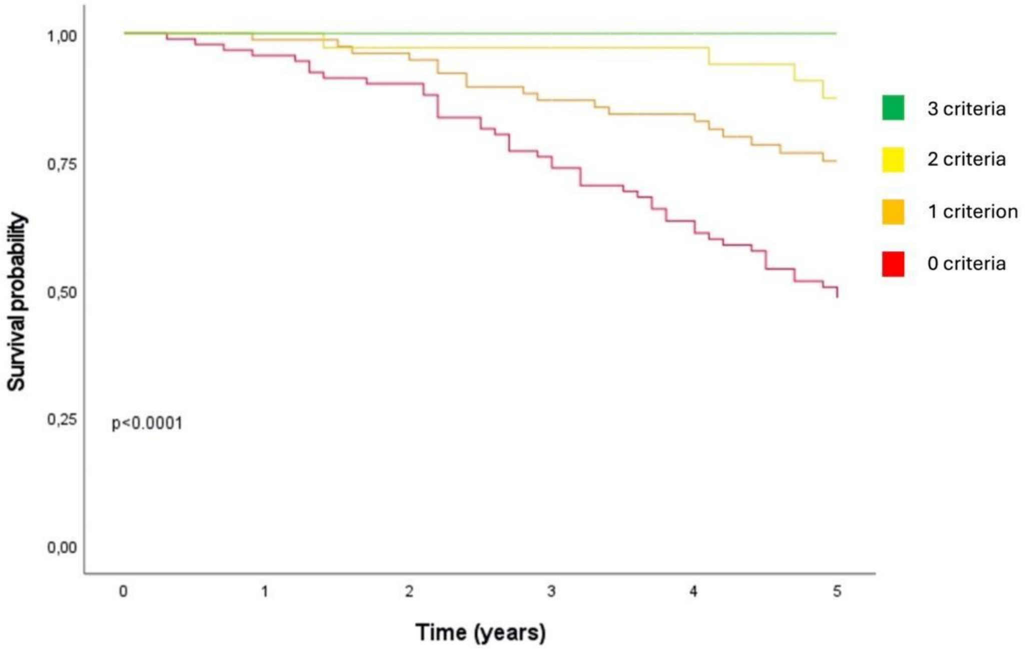

Patients at risk						
0 criteria	92	88	82	69	54	43
1 criterion	78	77	74	66	60	47
2 criteria	38	36	34	33	30	26
3 criteria	36	34	33	33	30	27

Figure S4. Transplant-free survival according to the number of low-risk criteria (BNP <50 ng/L, WHO FC I-II and a 6MWD >440 m).

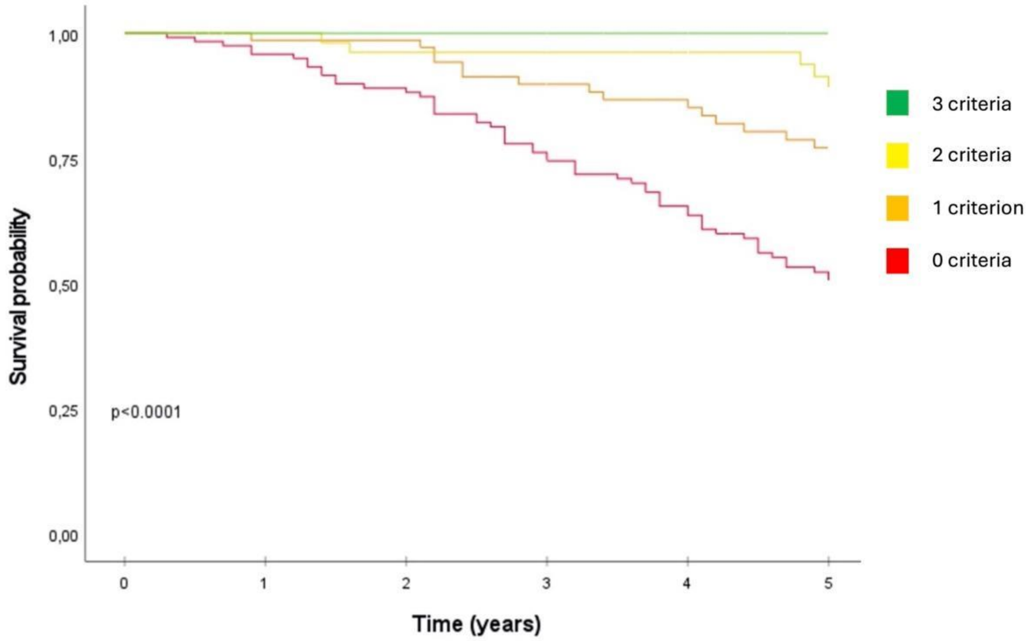

Patients at risk						
0 criteria	119	114	104	89	71	54
1 criterion	70	69	69	61	55	48
2 criteria	56	52	48	48	45	38
3 criteria	17	17	17	17	15	13

Figure S5. Long-term transplant-free survival according to the four-strata ESC/ERS^{6MWT} and ESC/ERS^{CPET} risk categories at follow-up.

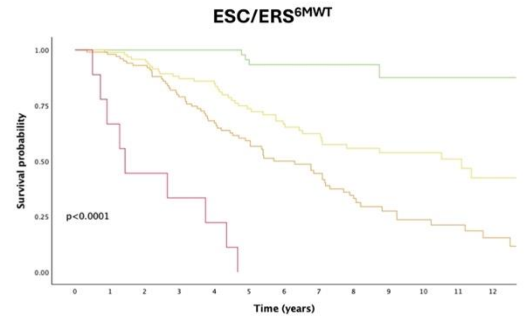

Patients at risk													
Low risk	57	54	52	52	49	43	31	27	20	15	11	9	9
Intermediate-low risk	95	93	90	81	73	59	48	42	33	27	21	18	9
Intermediate-high risk	101	99	92	79	62	51	37	32	24	14	11	9	5
High risk	9	6	4	3	2	0	0	0	0	0	0	0	0

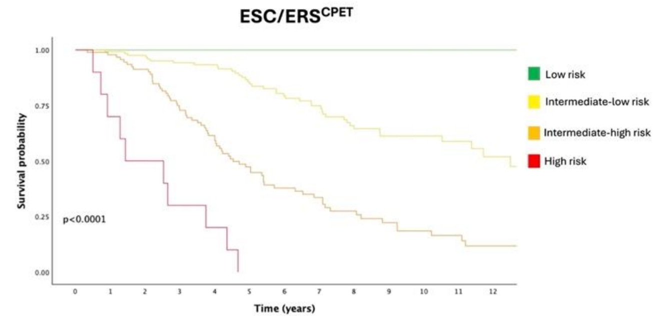

Patients at risk													
Low risk	34	32	31	31	28	25	16	12	9	8	5	4	4
Intermediate-low risk	126	123	118	112	103	89	73	66	50	36	29	24	14
Intermediate-high risk	92	90	84	69	53	39	27	23	18	12	9	8	5
High risk	10	7	5	3	2	0	0	0	0	0	0	0	0

Figure S6. Receiver operating characteristics (ROC) curves and areas under the curve (AUC) of the ESC/ERS^{6MWT} and ESC/ERS^{CPET} models for long-term survival.

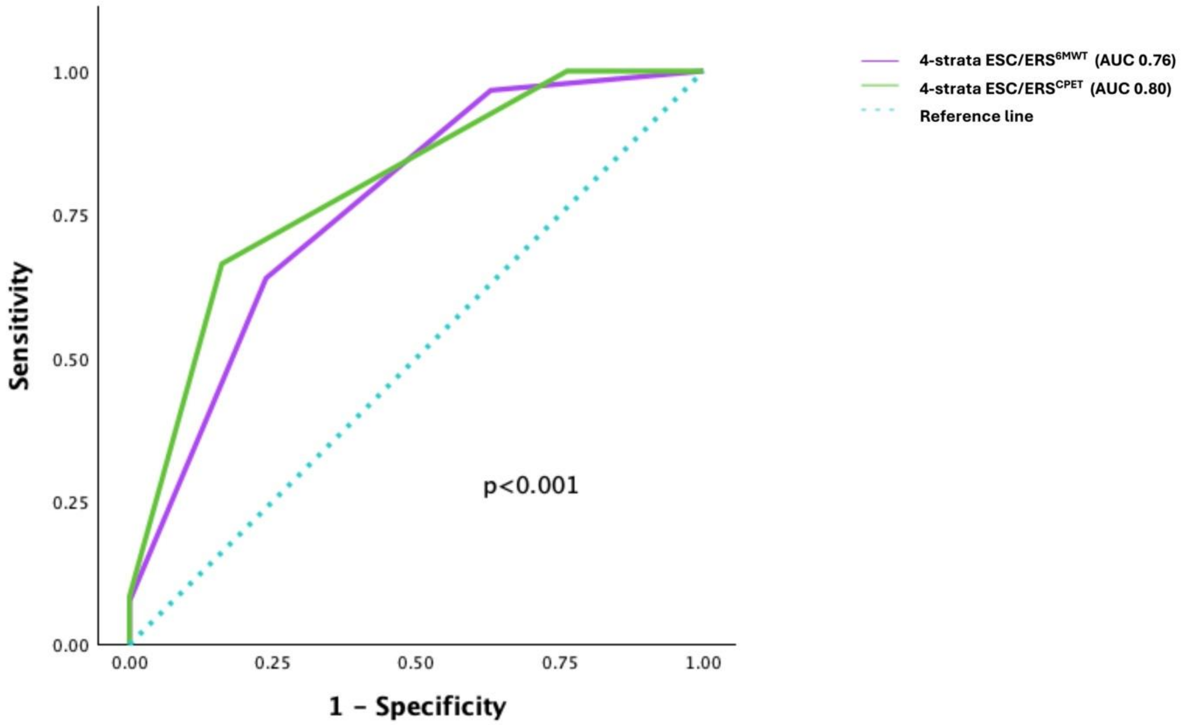

Supplementary figure S7. Transplant-free survival according to the four-strata ESC/ERS^{6MWT} and ESC/ERS^{CPET} risk categories at follow-up in patients with and without cardiopulmonary comorbidities.

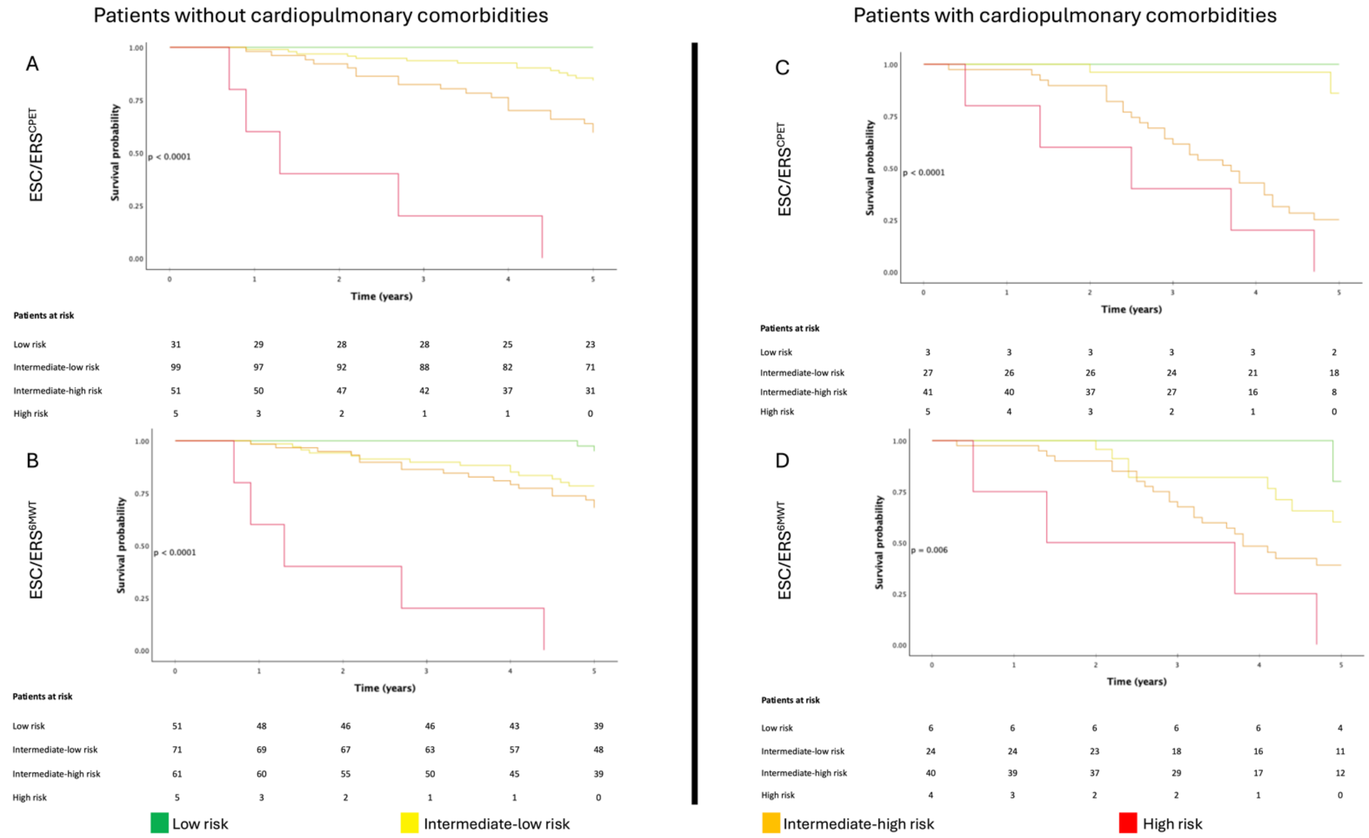

Supplement: Supplementary file 2 [file ERJ-02026-2024.Supplement.pdf]
